# Supplementary material for: Characterizing Longitudinal Antibody Responses in Recovered Individuals Following COVID-19 Infection and Single-Dose Vaccination: A Prospective Cohort Study
Source: Viruses. 2022 Oct 31;14(11):2416. doi: 10.3390/v14112416 (PMC9694471; doi:10.3390/v14112416)
Supplement: Supplementary file 1 [file viruses-14-02416-s001.zip › viruses-1952680-supplementary.pdf]

Supplemental Materials:

Table S1. Summary of serological test results.

**Test**	**No. samples tested**	**No. samples positive (%)**	**No. (%) positive <6 months**	**No.% positive >6 months**	***P-Value**
Total anti-RBD[$](Siemens)	340	324 (95%)	(191/196) 97%	(133/144) 92%	0.05
Anti-N IgG (Abbott)	335	158 (47%)	(140/194) 72%	(18/141) 13%	<0.001
Total anti-S (Ortho)	181	181 (100%)	(56/56) 100%	(125/125) 100%	--
Anti-RBD[$] IgG (MSD)	339	324 (96%)	(189/196) 96%	(135/143) 94%	0.53
Anti-N IgG (MSD)	339	196 (58%)	(149/196) 76%	(47/143) 33%	<0.001
Anti-S IgG (MSD)	339	303 (89%)	(181/196) 92%	(122/143) 85%	0.06

[$] 2 samples negative by anti-RBD (Siemens) were not available for Abbott or Ortho testing

* P-Values are reported for a Chi-square test (χ^2test) for two independent proportions.

Table S2. Descriptive summary of variables not included in the analysis for n= 21 vaccinated participants.

Count	Participant ID	Date of PCR Diagnosis	Viral Variant (Pangolin Lineage)	Vaccination Date	Diagnosis to Vaccination Time (Days)	Asymptomatic Infection	Febrile	Hospitalized
1	113	2021-01-11	B.1.2	2021-01-31	20	Yes	No	No
2	115	2020-12-09	B.1.36	2021-03-14	95	No	No	No
3	120	2020-12-10	B.1.2	2021-03-31	111	No	No	No
4	121	2020-12-14	P.2	2021-04-13	120	No	Yes	No
5	122	2020-12-02	B.1.36	2021-05-06	155	No	No	No
6	123	2020-12-14	B.1.1.231	2021-05-13	150	No	No	No
7	125	2021-02-03	--	2021-04-27	83	No	No	Yes
8	29	2020-10-25	--	2021-01-31	98	--	Yes	No
9	55	2020-03-22	A.1	2021-03-04	347	--	Yes	No
10	6	2020-10-02	B.1.128	2021-01-20	110	No	No	No
11	62	2020-03-09	--	2021-03-18	374	No	No	No
12	75	2020-03-23	A.1	2021-03-04	346	No	No	No
13	76	2020-05-01	B.1	2021-03-05	308	No	No	No
14	77	2020-04-01	B.1	2021-03-02	335	No	No	No
15	78	2020-03-20	B.1	2021-03-09	354	--	Yes	No
16	83	2020-03-20	A.1	2021-02-25	342	No	No	No
17	85	2020-03-30	B.1	2021-03-05	340	No	No	No
18	89	2020-03-23	A.1	2021-02-25	339	No	No	No
19	9	2020-05-06	--	2021-01-22	261	No	No	No
20	94	2020-08-24	B.1.128	2021-02-23	183	No	No	No
21	98	2021-01-01	B.1.36	2021-03-10	68	No	No	No

-- Indicates missing data, variables with missing data were not included in the statistical analysis and are presented for description and context.

Table S3. Descriptive summary of variables not included in the analysis for n= 21 unvaccinated participants.

Count	Participant ID	Date of PCR Diagnosis	Viral Variant (Pangolin Lineage)	Vaccination Date	Diagnosis to Vaccination Time (Days)	Asymptomatic Infection	Febrile	Hospitalized
1	124	2020-09-13	AE.8	-	-	No	No	No
2	131	2020-09-17	--	-	-	No	No	No
3	132	2020-07-11	--	-	-	No	No	No
4	136	2020-05-05	--	-	-	Yes	No	No
5	138	2020-12-26	--	-	-	Yes	No	No
6	18	2020-11-02	B.1.1.464	-	-	--	Yes	No
7	22	2020-08-08	B.1.422	-	-	Yes	No	No
8	34	2020-11-15	B.1.1.231	-	-	No	No	No
9	36	2020-08-09	--	-	-	No	No	No
10	37	2020-08-13	B.1.422	-	-	No	No	No
11	39	2020-10-30	--	-	-	No	No	No
12	42	2020-08-14	B.1.422	-	-	No	No	No
13	47	2020-08-03	--	-	-	--	Yes	No
14	5	2020-09-19	--	-	-	No	No	Yes
15	51	2020-11-08	B.1.36	-	-	No	No	Yes
16	52	2020-10-22	B.1.36	-	-	--	No	No
17	54	2020-11-19	B.1.36	-	-	No	Yes	Yes
18	61	2020-03-18	--	-	-	No	No	No
19	67	2020-05-01	--	-	-	No	No	No
20	79	2020-09-16	AE.8	-	-	No	No	No
21	8	2020-09-13	AE.8	-	-	No	No	No

- Participants were not vaccinated during the study period therefore they do not have a vaccination date and time from diagnosis to vaccination cannot be calculated.

-- Indicates missing data, variables with missing data were not included in the statistical analysis and are presented for description and context.

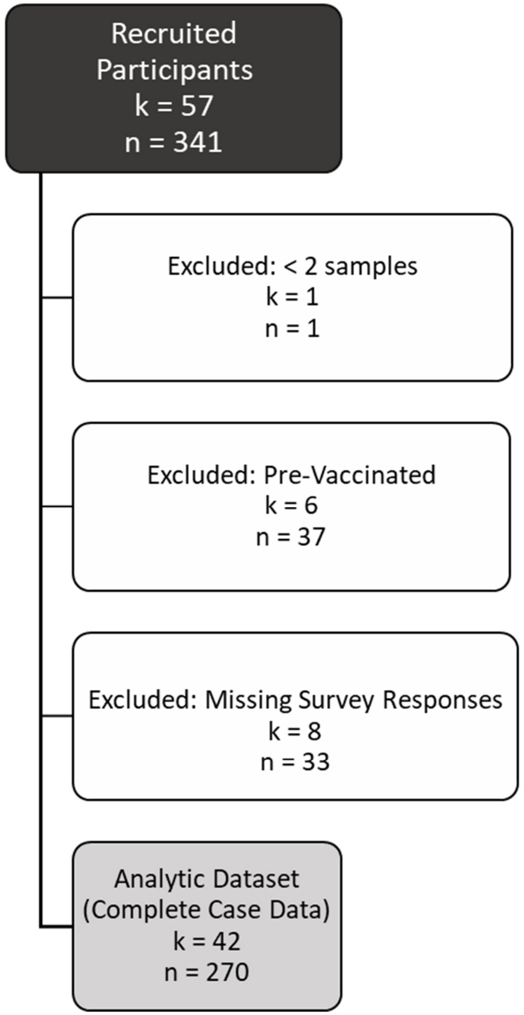

Figure S1. Exclusion criteria were applied to select an analytic data of n = 270 observations from k = 42 dependent participants (clusters).

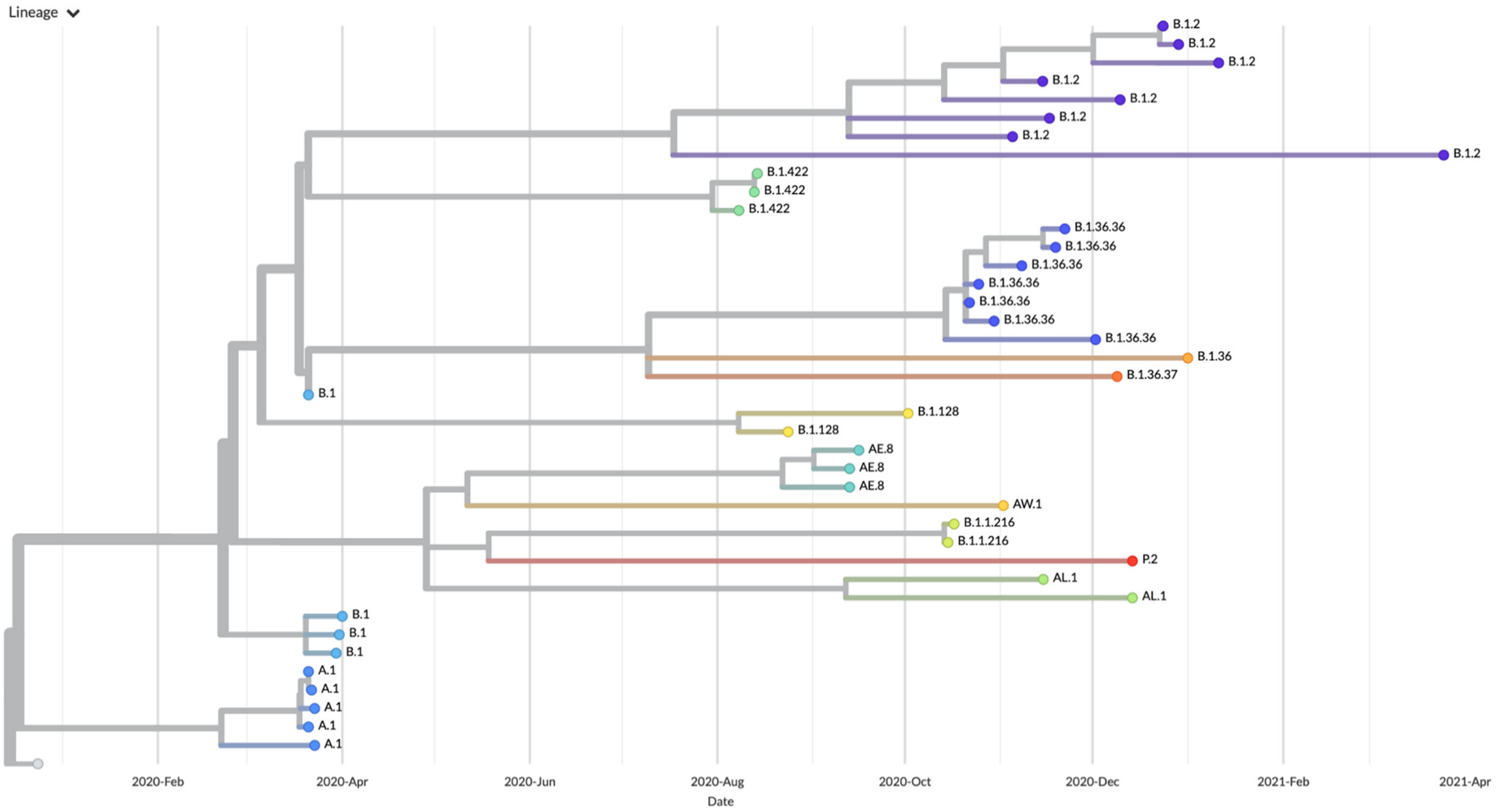

Figure S2. Phylogenetic analysis of CARE Study Participants' SARS-CoV-2 genomes. A phylogenetic tree was constructed using Fasttree and visualized in Nextstrain and lineage assignment was performed using the Phylogenetic Assignment of Named Global Outbreak Lineages tool (Pango/Usher Version 1.15.1), the Wuhan-Hu-1 isolate was used as the reference sequence (MN908947.3). Whole genome sequencing was performed at the BCCDC PHL using a modified ARTIC tiled 1200bp amplicon scheme, only sequences of sufficient quality were used in phylogenetic analysis (85% completeness and 10X depth of coverage). Date (x-axis) refers to the collection date of the positive SARS-CoV-2 diagnostic specimen. Colours on the tree tips correspond with SARS-CoV-2 lineage.
